# The effect of indomethacin on the muscarinic induced contractions in the isolated normal guinea pig urinary bladder

**DOI:** 10.1186/1471-2490-13-8

**Published:** 2013-02-07

**Authors:** Mohammad S Rahnama’i, Gommert A van Koeveringe, Philip EV van Kerrebroeck, Stefan GG de Wachter

**Affiliations:** 1 Department of Urology, Maastricht University Medical Centre, PO Box 5800, Maastricht, 6202, AZ, The Netherlands; 2 European Graduate School of Neuroscience, The Department of Psychiatry and Neuro psychology, Maastricht University, PO Box 616, Maastricht, 6200, MD, The Netherlands

## Abstract

**Background:**

To investigate the effect of prostaglandin depletion by means of COX-inhibition on cholinergic enhanced spontaneous contractions.

**Methods:**

The urethra and bladder of 9 male guinea pigs (weight 270–300 g) were removed and placed in an organ bath with Krebs’ solution. A catheter was passed through the urethra through which the intravesical pressure was measured. The muscarinic agonist arecaidine, the non-selective COX inhibitor indomethacin, and PGE_2_ were subsequently added to the organ bath. The initial average frequency and amplitude of spontaneous contractions in the first 2 minutes after arecaidine application were labelled F_ini_ and P_ini_, respectively. The steady state frequency (F_steady_) and amplitude (P_steady_) were defined as the average frequency and amplitude during the 5 minutes before the next wash out.

**Results:**

Application of 1 μM PGE_2_ increased the amplitude of spontaneous contractions without affecting frequency. 10 μM of indomethacin reduced amplitude but not frequency.

The addition of indomethacin did not alter F_ini_ after the first application (p = 0.7665). However, after the second wash, F_ini_ was decreased (p = 0.0005). F_steady_, P_steady_ and P_ini_ were not significantly different in any of the conditions. These effects of indomethacin were reversible by PGE_2_ addition._._

**Conclusions:**

Blocking PG synthesis decreased the cholinergically stimulated autonomous contractions in the isolated bladder. This suggests that PG could modify normal cholinergically evoked response. A combination of drugs inhibiting muscarinic receptors and PG function or production can then become an interesting focus of research on a treatment for overactive bladder syndrome.

## Background

The overactive bladder syndrome (OAB) is defined as urinary urgency with or without urgency incontinence, urinary frequency and nocturia. These symptoms still present a therapeutic challenge. Currently, antimuscarinic drugs are first-line treatment for OAB. How their beneficial action is achieved is still a matter of discussion. Depending on the studied compound, antimuscarinic drugs often have only moderate response rates when compared to placebo [1]. However, adverse effects and decreasing efficacy cause long-term compliance problems [2]. Therefore, it is desirable that alternative treatment methods are developed and made available.

The lower urinary tract has two basic functions: to store urine for most of the time at low pressure and expel it at a socially convenient time and place. Therefore, it is equipped with an extensive relay network to transmit information on bladder fullness to the brain [3]. One of the proposed mechano-transduction mechanisms is stretch dependent urothelial release of mediators such as acetylcholine, Nitric oxide, ATP and Prostaglandins (PG) [4]. PGE_2_ appears to be the main PG involved in the regulation of the bladder [5] and exert its effect through the endoprostanoid receptors, of which four subtypes (EP_1_, EP_2_, EP_3_, and EP_4_) have been described [6,7]. In the bladder, PG release depends on *de novo* synthesis rather than release from pre-formed stores [8]. Cyclooxygenase type 1 and 2 (COX-1 and COX-2) are the central enzymes in the production of PG [9]. COX-1 is a constitutive form, whereas COX-2 an inducible form in the bladder. Its expression is regulated by various stimuli, including pro-inflammatory cytokines and growth factors [9]. An increased expression of COX-2 has been described immediately after experimentally induced bladder outlet obstruction [10]. There is an increasing amount of data available pointing to a role of PG in the regulation of non-voiding contractions and afferent activity [10-13]. The isolated whole bladder shows autonomous small contractions, which resemble non-voiding contractions that increase in amplitude and/or frequency by muscarinic agonists [14,15] and PG [10]. Similarly, intravesical PG administration *in vivo* increases non-voiding contractions during bladder filling and decreases the inter-micturition interval, [16] whereas EP_1_ and EP_3_ knockout mice show an increased micturition threshold. Moreover, in these animals, the PGE_2_ induced hyperactivity is decreased [16]. How PG exerts its effect is not well understood. However, involvement of capsaicin sensitive afferents and autonomous ganglia has been suggested [12]. An interaction or crosslink between the cholinergic and prostanoid pathway has been suggested before [17] and may be another mechanism of action. This idea is supported by the fact that muscarinic agonists can induce production of PGE_2_[18].

The current study aims to further investigate the crosslink between the cholinergic and prostanoid pathway in order to explore a possible new treatment modality through COX inhibition for OAB. Therefore, the non-specific COX inhibitor indomethacin was used to investigate the effect of prostaglandin depletion on cholinergic enhanced spontaneous contractions.

## Methods

### Animals

A total of 9 male guinea pigs (weight 270–300 g) were killed by cervical dislocation, followed by exsanguination. Male guinea pigs were used because of the favourable urethral length, which made catheterization of the isolated bladder easier. All procedures were carried out with the approval of guidelines of the animal ethics committee of the University of Maastricht and were in line with European Community guidelines.

### Pressure recordings

The urinary bladder and proximal urethra were excised immediately after cervical dislocation of the animal and placed in Krebs’ solution (mM: NaCl 121.1; KCl 1.87; CaCl_2_ 1.2; MgSO_4_ 1.15; NaHCO_3_ 25; KH_2_PO_4_ 1.17; glucose 11.0), bubbled with 5% CO_2_ and 95% O_2_ (pH 7.4, 34°C). The urethra was cannulated with a flexible plastic cannula (2 mm diameter) secured using a fine ligature. The bladder was then transferred to a heated organ bath (40 mL, 33–36°C) containing constantly gassed Krebs’ solution, and the cannula was connected through a fluid-filled tube containing three-way connector to a pressure transducer (DTX Plus, Becton Dickinson, Franklin Lakes, NJ, USA). The bladder was allowed to empty. Subsequently, the bladder was filled to 2 ml in approximately 30 seconds. The transducer output was amplified, digitized at 20 Hz and recorded using a data capture system (MP100 with AcqKnowledge 3.7.3 software, BIOPAC systems inc, California). The pressure range of this apparatus was 0.02–180 cmH2O. The transducer was calibrated before each experiment. Recordings started 30 minutes after the animal was sacrificed.

**Figure 1:**
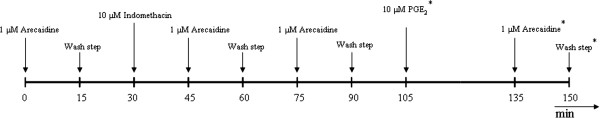
**The timeline of the experiments.** Pressure recordings started with stimulation of the guinea pig bladder by adding the cholinergic agonist arecaidine to the organ bath with Krebs’ solution in which the isolated guinea pig bladder was kept. After 15 minutes the Krebs’ solution is renewed (wash step). The COX inhibitor indomethacin was added 15 minutes after the first wash step. This was then followed by two sets of arecaidine stimulation and wash steps each separated by 15 minutes. In four experiments PGE_2_ was added to the organ 15 minutes after the third wash step which was then followed by a set of arecaidine stimulation and wash step, 30 minutes later.

The timeline of the experiments is given in Figure 1. The pressure recordings started with cholinergic stimulation with 1 μM of the cholinergic agonist, arecaidine (t = 0). This was followed by empting the organ bath and washing the bladder twice with fresh Krebs’ solution, 15 minutes after arecaidine stimulation (t = 15). In order to investigate the effect of inhibition of PG production on the arecaidine response, the non-selective COX inhibitor, indomethacin was applied to the bladder For this purpose, after another 15 minutes, 10 μM of the non-specific COX inhibitor indomethacin was added to the Krebs’ solution in the organ bath (t = 30), followed by a second stimulation with 1 μM arecaidine, given 15 minutes later (t = 45). After another 15 minutes, a wash step was conducted (t = 60). This was then followed by an arecaidine stimulation (t = 75), followed by a wash step, 15 minutes later (t = 90). At this point, in 4 experiments, 1 μM PGE_2_ was added to the organ bath after 10 minutes in order to check the reversibility of the indomethacin effect by PGE_2._ (t = 105). Again this was followed by arecaidine stimulation 30 minutes later (t = 135). At the end another wash step was conducted (t = 150).

### Drugs

Concentrated drug solutions were added directly to the bath to achieve the required final dilution. All drugs were added to the solution bathing the serosal surface. The non selective COX inhibitor, indomethacin (Sigma, St Louis, Missouri) was used in a concentration of 10 μM. As muscarinic agonist, a 1 μM concentration of arecaidine but-2-ynyl ester tosylate (Tocris, Avonmouth, UK) was used [14]. PGE_2_ (Sigma, St Louis, Missouri) was used in a concentration of 1 μM [19].

### Definitions

**Figure 2:**
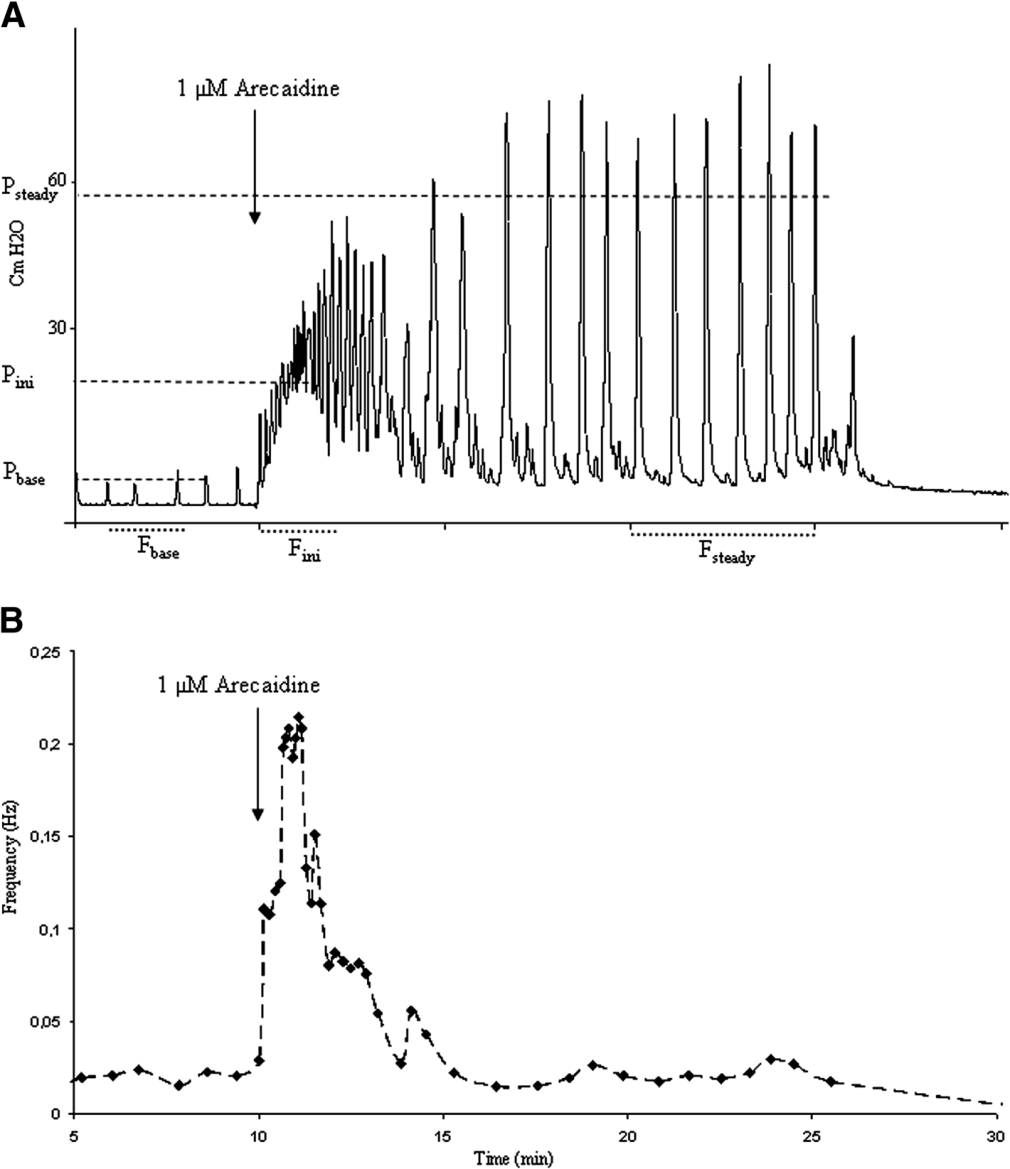
**Pressure changes and instantaneous frequency plot of an isolated guinea pig bladder induced by arecaidine. A**. Pressure changes of an isolated guinea pig bladder induced by 1 μM arecaidine. The average frequency in the initial phase, defined as the first 2 minutes after arecaidine application, is labelled F_ini_. The steady state frequency (F_steady_) is defined as the average frequency over a 5 minutes period prior to the wash out. **B**. Instantaneous frequency plot of an isolated guinea pig bladder. The time of stimulation with 1 μM arecaidine is indicated with an arrow. The frequency of the autonomous contractions initially increases and then return back to a lower steady level after approximately 5 minutes.

In Figure 2 the different defined phases of the bladder contraction amplitude and frequency are marked. The baseline activity (base) is defined as spontaneous baseline contractions before arecaidine stimulation. The initial phase (ini) is defined as the first 2 minutes after arecaidine application and is characterized by average frequency (F_ini_) and amplitude (P_ini_) of spontaneous contractions.The steady state (steady) is defined as the 5 minutes before the next wash out, and is characterized by frequency (F_steady_) and amplitude (P_steady_).

### Instantaneous frequency plot

The raw pressure at the peak of every contraction was acquired from the measurement system along with the exact time of the contractions. The frequencies were then calculated and plotted as demonstrated in the figures.

### Statistical analysis

The analysis of the data was done, by conducting a double sided, paired t-test using the SPSS software.

## Results

The figures displayed represent raw experimental data conducted in different animals on different days.

### Response to muscarinic stimulation

The isolated bladder response to the non-selective muscarinic agonist, arecaidine, is shown in Figure 2 (arrow). The initial phase was characterized by a rapid rise in basal pressure, followed by high-frequency bladder contractions. Frequency declined within 5 minutes after which contraction amplitude increased, eventually reaching a steady state. These findings are consistent with previous observations [15,20].

### Effect of prostaglandin and COX inhibition on the baseline activity

**Figure 3:**
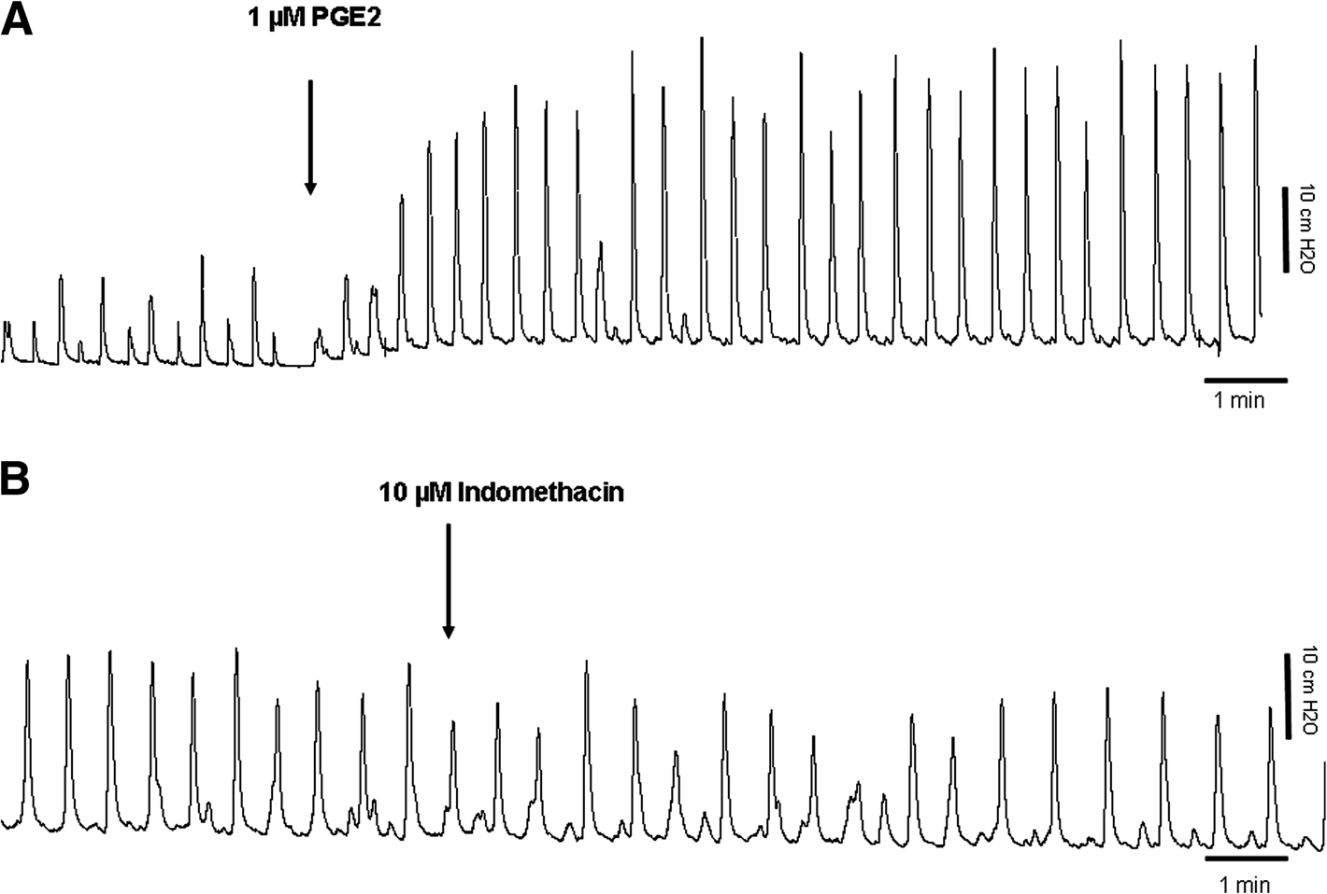
**Spontaneous activity of the isolated guinea pig bladder after administration of PGE**_**2 **_**and indomethacin. A**. Spontaneous activity of the isolated guinea pig bladder. The arrow indicates when 1 μM of PGE_2_ is added. The amplitude of the contractions is significantly increased. **B**. Spontaneous activity of the isolated guinea pig bladder. The arrow indicates when 10 μM indomethacin is added. The amplitude of the contractions is slightly decreased by indomethacin. However, the frequency of contractions is not affected.

The effects of PGE_2_ and indomethacin on the baseline transients are shown in Figure 3. P_base_ increased about 2-fold in the presence of 1 μM PGE_2_ (control 8.0 ± 4.6 cmH2O vs. PGE_2_ 17.1 ± 7.5 cmH2O; p = 0.01; n = 5). F_base_ was not affected by PGE_2_ (control 0.026 ± 0.01 Hz vs. PGE_2_ 0.039 ± 0.02 Hz; p = 0.26; n = 5).

Application of 10 μM of indomethacin decreased P_base_ (control 13.2 ± 6.2 cmH2O vs. indomethacin 11.6 ± 5.9 cmH2O; p = 0.03; n = 6). F_base_ was not affected by indomethacin (control 0.028 ± 0.0066 Hz vs. indomethacin 0.030 ± 0.0051 Hz; p = 0.32; n = 6).

### Effect of indomethacin and PGE_2_ on the muscarinic response

**Figure 4:**
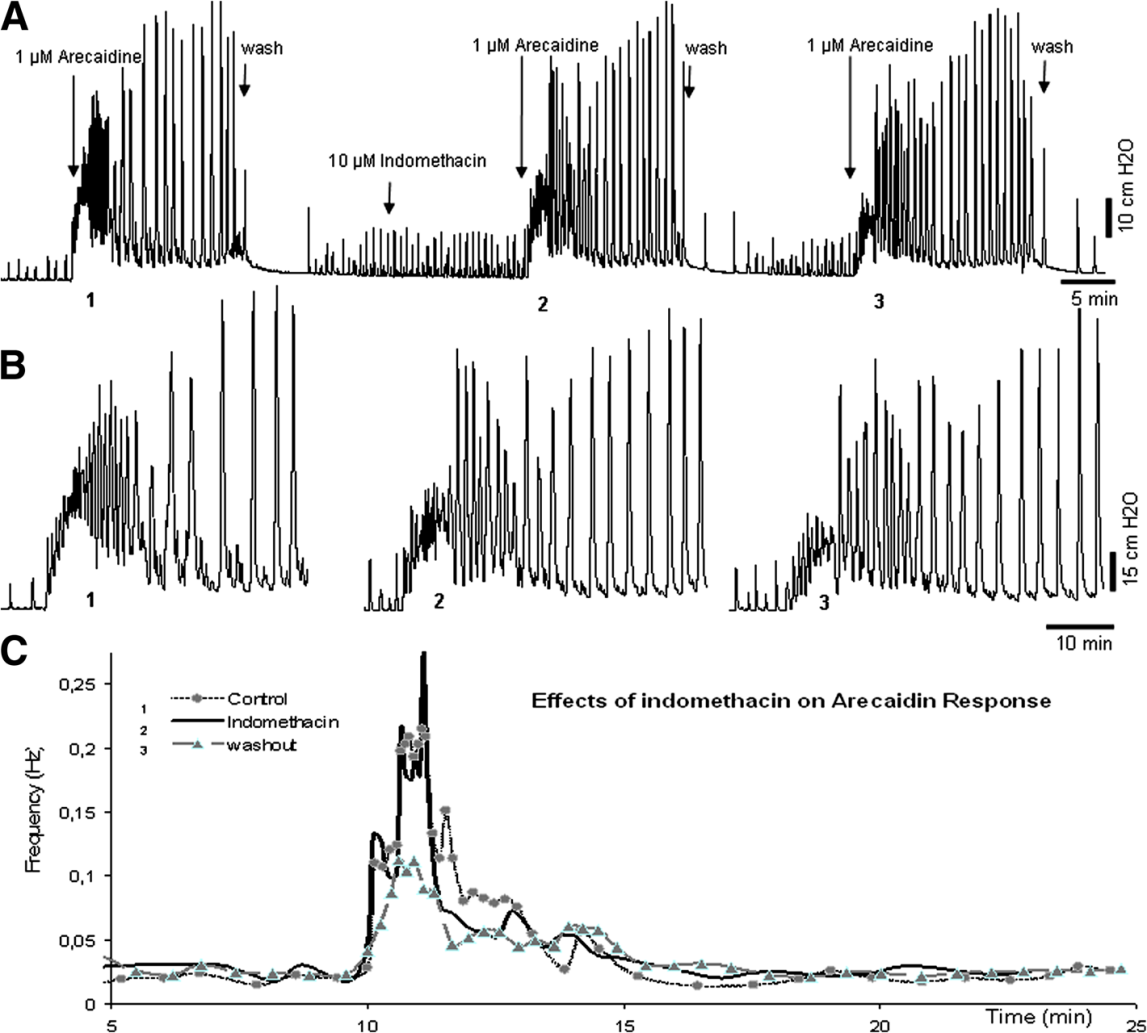
**Timeline, pressure changes and instantaneous frequency plot of an isolated guinea pig bladder pre-exposed to indomethacin before stimulation with arecaidine. A**. an outline of the experiment. After the first 1 μM arecaidine simulation (enlarged in panel B as 1), the organ bath is washed to remove the arecaidine. After 15 minutes, 10 μM indomethacin is added followed by a second stimulation of arecaidine (enlarged in panel B as 2). After a wash step, a third arecaidine stimulation is performed (enlarged in panel B as 3). **B**. Parts of panel A in an expanded scale. **C**. Instantaneous frequency plot comparing the three conditions described in panel A.

The recordings of an experiment with COX inhibitor indomethacin are shown in Figure 4. At t = 30 indomethacin was added to the bath. The bladder response on arecaidine stimulation in the presence of indomethacin (t = 45) was not different from the response to arecaidine stimulation in the absence of indomethacin (t = 0), for any of the parameters P_ini_ (p = 0.081), F_ini_ (p = 0.77), P_steady_ (p = 0.19), F _steady_ (p = 0.32).

However, after a wash step and a the second stimulation, with arecaidine (t = 75), F_ini_ was significantly decreased (p = 0.0005), whereas P_ini_ (p = 0.086), F_steady_ (P = 0.32) and P_steady_ (p = 0.33) remained unchanged.

**Figure 5:**
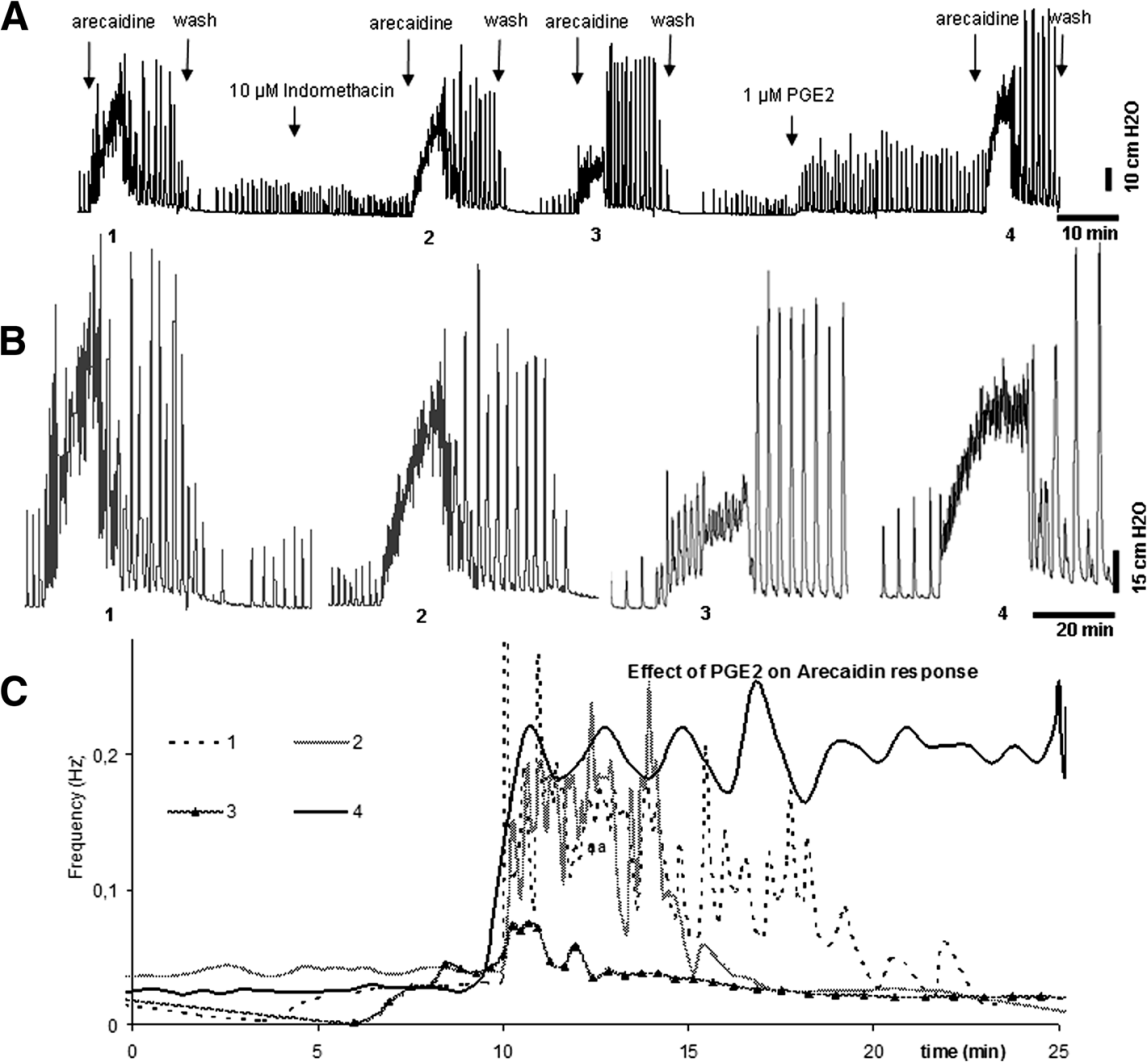
**Timeline, pressure changes and instantaneous frequency plot of an isolated guinea pig bladder after stimulation with arecaidine showing the reversibility of the indomethacin effect by PGE**_**2**_**. A**. an outline of another typical experiment. The same protocol as described in Figure 3 was followed. To check the reversibility, at the end of the experiment, 1 μM PGE_2_ is added in to the organ bath and a last arecaidine stimulation is conducted. The first arecaidine stimulation is marked as (1) followed by the stimulation with presence of indomethacin (2) followed by stimulation after a wash step (3). At the end an arecaidine stimulation is shown after adding PGE_2_ to the system (4). **B**. Each of the arecaidine responses shown in panel A in an enlarged scale, starting from the left, the first trace shows the basic arecaidine response (1), the second trace shows the previously shown effect of the inhibition of PG production (2). The third trace shows the effect of washing the organ bath with fresh Krebs’ before stimulation with arecaidine (3). The last trace from the right shows the effect of addition of exogenous PGE_2_ (4). **C**. Instantaneous frequency plot comparing the four conditions described in panel A.

To check the reversibility of the described indomethacin effect after the wash step, by prostaglandin, PGE_2_ was given exogenously at t = 105 in four animals. One of these recordings is shown in Figure 5.

After adding PGE_2_ a strong response, i.e. high amplitude contractions, were observed as shown in Figure 5. In order to achieve a steady state before adding arecaidine, arecaidine was added 30 minutes later instead of 15 minutes.

Data of all four experiments show that, compared to arecaidine stimulation after indomethacin exposure (t = 45 and T = 75), F_ini_ was increased (p = 0.014), so the indomethacin effect on F_ini_ was reversed by PGE_2_. There were no changes noted in P_ini_ (0.38), neither F_steady_ (0.27) nor P_steady_ (0.19). F_ini_ at t = 135 was not significantly changed when compared to the initial arecaidine stimulation (t = 0) (p = 0.06).

## Discussion

Presence of PG receptors in the bladder and involvement of PG in the bladder physiology has been demonstrated before [6,13,21,22]. The urothelium is shown to release acetylcholine, ATP, Nitric Oxide and prostaglandin [4,6]. The physiological role of these signals is poorly understood and a possible interaction between them has not widely been considered.

The link between PG and cholinergic effects in the bladder has been suggested before. In a study in rat detrusor it was shown that indomethacin suppressed frequency of muscarinic-induced contractions but only in strips of certain orientation and with urothelium intact [23]. Furthermore, in another study in rat bladders studied in an organ bath model it was shown that PGE_2_ release could be antagonized by indomethacin and concluded that COX inhibitors suppress ATP release from bladder epithelium via decreasing PGE_2_. In addition, EP1 and/or EP3 receptors were suggested to participate in this effect [24]. In a study in rabbit detrusor strips it was shown that both indomethacin and ibuprofen suppress spontaneous contractions [25]. In addition, it has been suggested that endogenous PGs in isolated human detrusor facilitate the action of acetylcholine [17]. This effect was thought to be through an increase in the concentration of the cholinergic neurotransmitter probably via an inhibition of the acetylcholinesterase activity [17]. Moreover, PG has been suggested to have a modulatory role in the release of acetylcholine and ATP in the guinea pig bladder [26].

The aim of our study was to further investigate the link between the prostaglandin and the cholinergic system and explore the possibility of COX inhibition as a treatment modality for OAB. It has been demonstrated that that there is a basal acetylcholine release in human detrusor muscle in vitro [27]. This release was resistant to nerve blocker tetrodotoxin, and considerably reduced when the urothelium was removed, suggesting that the released acetylcholine was of non-neuronal origin and, at least partly, generated by the urothelium. Stretch of the muscle increased the release. There are thus reasons to believe that acetylcholine can be generated by non-neuronal structures within the bladder, and it may be speculated that urothelially derived acetylcholine can excite suburothelial afferent nerves and that this contributes to OAB. During filling of the bladder, there is no activity in the parasympathetic nerves innervating the bladder, and direct evidence for spontaneous release of acetylcholine from nerves during the filling phase of the bladder has so far been lacking [28]. However, it has been shown that there is a spontaneous tetrodotoxin-resistant release of acetylcholine from autonomic cholinergic nerves in guinea pig and rat bladders under both in vitro and in vivo conditions [29]. This release was shown to significantly affect bladder contractility (autonomous activity) [29]. These findings provide further support for the hypothesis that antimuscarinic drugs can act also by inhibiting a myogenic afferent pathway during the filling phase [28]. Thus, during the storage phase, acetylcholine may be released from both neuronal and non-neuronal sources, and directly or indirectly excite afferent nerves suburothelially and within the detrusor.

It is has been shown that an isolated bladder in an organ bath shows spontaneous rhythmical contractions [14,15]. The frequency of this baseline activity was shown to be unaffected by PGE_2._ and the COX-inhibitor indomethacin (Figure 3). Although F_base_ was not significantly affected by PGE_2_ (control 0.026 ± 0.01 Hz vs. PGE_2_ 0.039 ± 0.02 Hz; p = 0.26; n = 5) there was an increase of about 50%. Therefore, this might be due to a lack of power in our study and might become significant if more animals were tested. On the other hand, the amplitude of the baseline contractions was significantly raised by PGE_2_ and decreased by indomethacin, indicating that PG is needed for and has a stimulating effect on the amplitude of these baseline contractions.

The non-selective muscarinic agonist, arecaidine induces a rapid rise in basal pressure with high-frequency bladder contractions which declined within 5 minutes. After this, contraction amplitude increased, eventually reaching a steady state. These findings are consistent with previous observations [15,20] and represent a typical response by the isolated bladder to arecaidine as shown in Figure 2.

Furthermore, data presented in this paper show that inhibition of PG production by indomethacin diminished the average frequency of the initial burst of transients of the isolated guinea pig bladder, in the first 2 minutes after of the application of the muscarinic agonist, arecaidine (F_ini_).

As explained in Figure 2B, indomethacin by itself, i.e. in the absence of arecaidine, did not change baseline contraction frequency but slightly reduced contraction baseline amplitude. However, arecaidine stimulation after COX inhibition by indomethacin application, lead to a significantly less outspoken response, after the second arecaidine stimulation. The explanation of this delayed effect of indomethacin on F_ini_ could simply be due to a longer incubation time needed for indomethacin to exert its effect. Indomethacin is still believed to be present in the organ bath since the recordings after the second wash step are not the same as control. This can be clearly seen in Figures 4 and 5, especially at the diagram at the bottom of the figures. The difference in 1 (control) and 3 (after second wash step) is only in the addition of indomethacin and since these two recordings are persistently different in all experiments we assume that indomethacin is still present in the system. Furthermore, the conducted control experiments (data not included) have ruled out that this effect is simply due to time or a repeated wash step.

However, another plausible explanation may be that the second wash step, applied after the second arecaidine stimulation, could have caused a further washout of the previously produced PG. Since *de novo* production of the PG is inhibited by indomethacin, there is no new PG production in the isolated bladder. The delayed effect may therefore also be explained by the small amount of previously produced PG, which might still be present and active in the system during the first arecaidine stimulation. Therefore, the initial burst induced by arecaidine is not affected the first time. We hypothesize that after the addition of indomethacin followed by the second wash step, there is practically no PG left in the organ bath and the tissue. Therefore, there is a significantly less outspoken response to arecaidine.

Thus, inhibition of PG production seems to reduce F_ini_ of the muscarinic response and it can be suggested that PG is necessary for the normal increase in autonomous activity following cholinergic stimulation, i.e. the arecaidine response. In line with this, an exogenous dose of PGE_2_ increases the initial burst frequency after arecaidine stimulation, to levels even higher than the control (Figure 4).

PG has been suggested to be linked to acetylcholine before. In a study conducted in another species and another organ system, namely the mouse mesenteric artery, it has been shown that acetylcholine hyperpolarises the smooth muscle cell membrane with two kinetic components, one which is indomethacin-sensitive and the other which is indomethacin-insensitive [30]. The indomethacin-sensitive component is suggested to be linked to prostanoids [30]. Thus, PG was suggested to have a role in this muscarinic response of these arterial smooth muscle cells, which is confirmed by our experiments. Moreover, it is known that in the guinea pig bladder, ATP can activate PGE_2_ production by a complex mechanism involving the purinergic receptors P2X and P2Y [13]. This ATP response was shown to be inhibited by the COX inhibitor indomethacin [13]. This suggests a complex mechanism of cholinergic receptor mediated, ATP induced, stimulation of COX enzyme resulting in an increased PG production.

Another clue for a link between PG and the muscarinic system comes from clinical data. It is known that inhibition of cholinergic activity in the bladder by antimuscarinic drugs, although effective in reducing the symptoms of urgency and frequency, does not have a high enough dosage to target the muscle cells [31,32]. Therefore, the suggestion is made that anticholinergic drugs could target the mechanisms operating during the filling phase [33-35] through locally produced substances such as ATP, NO and PG [10,36].

Drake et al. reported increased bladder sensation to be associated with localized contractile activity in the bladder wall of human subjects (micro-motions) [37]. These micro-motions are significantly more prevalent in patients with urgency than in asymptomatic volunteers [37]. In other words, urgency is suggested to be associated with autonomous activity of the detrusor and altered micro-motions.

It has been shown that patients with the overactive bladder syndrome (OAB) have an increased urinary PG [38,39] which is thought to affect bladder activity directly by effects on smooth muscle and/or indirectly via effects on neurotransmission [40].

## Conclusions

In summary, our data show that inhibition of PG production has an influence on the cholinergically induced bladder response in the isolated bladder. In order to evaluate the effect of PG on the autonomous activity in the presence of local reflex loops and control by the central nervous system, these experiments should be performed *in vivo*. By this, a combination of drugs inhibiting both muscarinic receptors and PG function or production can become an interesting focus of research in the quest for a better treatment for OAB. In order to gain a full understanding of the PG effect shown on the cholinergically induced bladder response, more studies need to be conducted using selective EP blockers in vitro and in vivo.

## Competing interest

The authors declare that they have no competing interests.

## Authors’ contribution

MSR carried out the experiments, analysed the data and drafted the manuscript. GVK helpt with the study design. SdW supervised the analysis of the data. GvK, SdW and PvK supervised the drafting of the manuscript. All authors read and approved the final manuscript.

## Pre-publication history

The pre-publication history for this paper can be accessed here:

http://www.biomedcentral.com/1471-2490/13/8/prepub
